# Composition of nutrients, heavy metals, polycyclic aromatic hydrocarbons and microbiological quality in processed small indigenous fish species from Ghana: Implications for food security

**DOI:** 10.1371/journal.pone.0242086

**Published:** 2020-11-12

**Authors:** Astrid Elise Hasselberg, Laura Wessels, Inger Aakre, Felix Reich, Amy Atter, Matilda Steiner-Asiedu, Samuel Amponsah, Johannes Pucher, Marian Kjellevold

**Affiliations:** 1 Institute of Marine Research, Bergen, Norway; 2 German Federal Institute for Risk Assessment, Berlin, Germany; 3 Council for Scientific and Industrial Research, Food Research Institute, Accra, Ghana; 4 Department of Nutrition and Food Science, School of Biological Sciences, University of Ghana, Legon, Accra, Ghana; 5 Department of Fisheries and Water Resources, University of Energy and Natural Resources, Sunyani, Ghana; Universidade de Sao Paulo, BRAZIL

## Abstract

The triple burden of malnutrition is an incessant issue in low- and middle-income countries, and fish has the potential to mitigate this burden. In Ghana fish is a central part of the diet, but data on nutrients and contaminants in processed indigenous fish species, that are often eaten whole, are missing. Samples of smoked, dried or salted *Engraulis encrasicolus* (European anchovy), *Brachydeuterus auritus* (bigeye grunt), *Sardinella aurita* (round sardinella), *Selene dorsalis* (African moonfish), *Sierrathrissa leonensis* (West African (WA) pygmy herring) and *Tilapia spp*. (tilapia) were collected from five different regions in Ghana. Samples were analyzed for nutrients (crude protein, fat, fatty acids, several vitamins, minerals, and trace elements), microbiological quality (microbial loads of total colony counts, *E*. *coli*, coliforms, and Salmonella), and contaminants (PAH4 and heavy metals). Except for tilapia, the processed small fish species had the potential to significantly contribute to the nutrient intakes of vitamins, minerals, and essential fatty acids. High levels of iron, mercury and lead were detected in certain fish samples, which calls for further research and identification of anthropogenic sources along the value chains. The total cell counts in all samples were acceptable; *Salmonella* was not detected in any sample and *E*. *coli* only in one sample. However, high numbers of coliform bacteria were found. PAH4 in smoked samples reached high concentrations up to 1,300 μg/kg, but in contrast salted tilapia samples had a range of PAH4 concentration of 1 μg/kg to 24 μg/kg. This endpoint oriented study provides data for the nutritional value of small processed fish as food in Ghana and also provides information about potential food safety hazards. Future research is needed to determine potential sources of contamination along the value chains in different regions, identify critical points, and develop applicable mitigation strategies to improve the quality and safety of processed small fish in Ghana.

## Introduction

With a growing global population and an increasing number of undernourished people, having access to sufficient amounts of safe and nutritious food is crucial [1]. The significance of food and nutrition security (FNS) is anchored in the United Nations Sustainable Development Goals (SDGs), specifically SDG 2 “Zero Hunger” and SDG 3 “Good Health and Well-Being” [2]. Fish is a source of many essential nutrients [3–7], and can substantially improve FNS especially in a diet that largely consists of starchy staple foods such as cassava, yam, rice, maize and millet, which is the case in Ghana [8–11]. Compared with animal source foods, starchy foods contain low amounts of micronutrients, have poorer protein quality and may be a source of phytate which further aggravates the severity of undernutrition by inhibiting the absorption of essential minerals and limiting protein and lipid utilization [12, 13]. Fish is an essential part of the Ghanaian diet and is accessible at a low cost, with an apparent per capita consumption of 28 kg/year and constituting 50–80% of consumed animal protein [14–16]. In Ghana small fish is usually eaten whole [3], which represents the most beneficial form of consuming fish in terms of nutrient density, providing vitamins A, D and B_12_, minerals such as calcium and trace elements iodine, selenium, iron and zinc [17, 18]. In contrast, when consuming larger fish, the micronutrient-rich organs, viscera and bones are usually removed, which limits the potential nutrient content [19].

Different processing techniques, such as smoking, drying and salting enables the availability of marine and freshwater fish throughout the country [20]. To prevent spoilage and prolong shelf life, smoking is the leading technique of fish processing in Ghana constituting 70–80% of locally consumed fish, while the remaining percentage is consumed fresh or subjected to drying, salting, frying or fermentation [21]. Commonly, a metal drum kiln or “Chorkor smoker” is used [22–24] in which fat and other fluids from fish may drip into the open flames and form harmful substances, such as polycyclic aromatic hydrocarbons (PAHs), which have reported carcinogenic and mutagenic properties [23, 25, 26]. Another potential hazard for consumers is microbial contamination, especially in countries where cold storage is not widely available to inhibit microbial growth. Here, the management and way of processing, like smoking or drying of the fish, plays an essential role [24, 27]. Further, improper handling and hygiene practices, storage, and insufficient smoking or drying can increase the likelihood of recontamination and growth of microorganisms including pathogenic bacteria from fecal sources [28–31]. Additionally, fish may be a source of heavy metals e.g. mercury, arsenic, lead and cadmium [32, 33]. The majority of fish consumed in Ghana is of marine origin and low levels of mercury have been registered in fillet of fresh marine fish from the Gulf of Guinea [34, 35]. Nonetheless, one of the largest e-waste recycling sites in Africa is located in Accra, resulting in large-scale environmental contamination with many toxicants, particularly lead, at high concentrations through smoke emission [36, 37]. The elevated ambient burden of heavy metals in surrounding areas contributes to the toxic exposure of the general population, and quite possibly, to the fish markets situated nearby [36]. In the freshwater lakes and rivers, discharge of effluents from gold mines and other industries increases the presence of mercury and other contaminants [38, 39].

The current study is part of the SmallFishFood project (https://smallfishfood.org/), an international consortium collaborating to understand how socio-cultural, economic and institutional transformations of the fish food and value chain—from ecosystem to consumer—can contribute to improved, sustainable utilization of small fish resources for Africa’s low-income population. Access to local, reliable and up-to-date food composition data (FCD), particularly on foods that are essential to vulnerable population groups, is scares but imperative to improve FNS. Although the main body of FCD comprises nutrients, data on potentially harmful food components such as heavy metals, polycyclic aromatic hydrocarbons (PAHs) and pathogenic microorganisms are also important for nutritional recommendations, as it provides the basis for the estimation of exposure, risk assessments and risk-benefit analysis [40]. Combined, this knowledge may also be used to develop programs and policies to improve FNS [41, 42]. In most published data on nutritional quality and safety parameters in fish only the flesh of raw fish has been analyzed, and the analytical data on nutritional quality and safety of processed whole small fish are scarce.

The aim of the present study was to generate analytical data on selected nutrients, as well as microbial, heavy metal and PAH contamination in six commercially important processed small fish species sampled from five regions in Ghana.

## Materials and method

### Sampling

Selection of fish species sampled from Ghana were based on a market survey conducted in February 2018, where open markets in Accra, Techiman and Kumasi were visited to determine the most frequent fish species, fish sizes and the most common processing method used for each species. During November 2018, samples of processed small marine fish species *Engraulis encrasicolus* (European anchovy), *Brachydeuterus auritus* (bigeye grunt), *Sardinella aurita* (round sardinella), *Selene dorsalis* (African moonfish) and small freshwater species *Sierrathrissa leonensis* (West African (WA) pygmy herring), and *Tilapia spp*. (tilapia) were sampled at selected fish markets throughout Ghana. The identification details of the sampled species, including scientific and Ghanaian names, are presented in Table 1. The species were sampled from six markets in five cities, representing five different regions in Ghana; Accra Agbogbloshie and Adabraka Market (Accra, Greater Accra region, near coast), Kumasi Central Market (Kumasi, Ashanti region, about 215 km from coastline), Techiman Market (Techiman, Brong-Ahafo region, about 335 km from coastline), Tamale Market (Tamale, Northern region, about 600 km from coastline) and Bolgatanga Market (Bolgatanga, Upper East region, about 760 km from coastline).

**Table 1:** Species, habitat, weight and length of fish sampled from five different locations in Ghana.

Common name	Scientific name	Local name	Habitat	Tissue analyzed	Fish length (cm)^a^	Processing method	Batches	Composite samples (n)	Specimens in each composite sample	Batch/ composite weight (g)
**European anchovy**	*Engraulis encrasicolus*	Amoni, Bonu, Abobi, Saskawesi, Ablobi^d^	Marine, pelagic	Whole fish^b^^,^^c^	6.3 (5–7.6)	Smoked	25	5	>250	100/500
**Bigeye grunt**	*Brachydeuterus auritus*	Boeboe, Moe, Hawui, Eboe, Ano kpetei^d^	Marine, benthopelagic	Whole fish without head^b^/whole fish^c^	12.4 (11.8–12.9)	Smoked	15	3	45 (42–50)	100/500
**African moonfish**	*Selene dorsalis*	Antele-wawaa, Ngogba lolotor, Epo edwire, Tantemire ansoradze, Epo edwile, Ndademire^d^	Marine, demersal	Whole fish^b^^,^^c^	9 (6.6–10.8)	Smoked	12	3	75 (35–100)	100/500
**Round Sardinella**	*Sardinella aurita*	Kankama, Man, Vetsimu, Eban^d^	Marine, pelagic	Whole fish^b^^,^^c^	13.3 (12.7–15.4)	Smoked	24	5	49 (33–73)	100/500
**Tilapia**	*Tilapia spp*.	Apatire Tidie, Akpaa, Apataa, Koobi^e^	Freshwater, benthopelagic	Whole fish without guts^b^/ whole fish^c^	11.3 (10.2–12.8)	Salted	23	5	43 (22–73)	100/500
**West African pygmy herring**	*Sierrathrissa leonensis*	One man thousand, Woevi^e^	Freshwater, pelagic	Whole fish^b^^,^^c^	4.6 (3.4–6)	Dried or smoked	7	3	230 (>100–500)	100/500

^a^ Numbers presented as mean and range (min-max).

^b^ Tissue analyzed for nutrients, heavy metals and PAHs.

^c^ Tissue analyzed for microbiological contamination.

^d^ Local names from [59].

^e^ Local names.

#### Sample size

The sampling was conducted according to Greenfield and Southgate’s requirements for retail sampling [43]. Sample size (number of specimens) was adapted from Öhrvik, von Malmborg [44], who estimated that each composite sample of fish should consist of minimum 12 specimens. The calculation was based on a formula for sample size by Greenfield and Southgate [43] using the variability of key nutrients in fish including docosahexaenoic acid (DHA) [45], vitamin D and selenium [46]. This would require that for each fish species, more than 60 individuals in total and 12 individuals from each selected location should be collected. However, approximately 500 g of organic material was estimated to be needed to perform the targeted analyses, and more than 12 individuals of each fish species were thus collected from each location.

#### Sampling procedure

Upon arrival at the fish markets, the sampling was started in one section of the market and the selected fish species were sampled from every third market stall. The selected species were identified by a taxonomist, and two separate batches of approximately 100 g of fish was weighed and collected in zip lock plastic bags. Depending on availability of the selected fish species at each fish market, which was influenced by the unpredictable nature of fish supply from small-scale fishermen and fishmongers, between 1–5 batches of each processed fish species were collected from different market stalls at each location. After sampling, the samples were stored in Styrofoam boxes and kept at room temperature along the sampling route from Bolgatanga to Accra. The sampling was conducted between the 2^nd^ and 24^th^ of November 2018. In Accra, the batches of each sampled fish species were pooled into composite samples for analysis of nutrients, heavy metals and PAHs, with each composite sample representing one species from one location. Subsequently, the pooled samples were shipped in boxes containing cooling elements via express airmail to Norway on November 29^th^, 2018. Un-pooled batch samples for microbial analysis were shipped via express airmail to Germany on December 17^th^, 2018.

### Chemical analyses of nutrients and contaminants

Depending on the fish species, the tissue for analysis was prepared according to Ghanaian food customs, and may or may not include head, viscera, bones, scales or other parts (Table 1). The composite samples were homogenized as per edible parts prior to analysis in a food processor (Braun 3210, Neu-Isenburg, Germany), and subsamples of the homogenate were distributed into tubes (Thermo Scientific Nunc A/S, Roskilde, Denmark) and stored at -20°C or -80°C pending analysis. Samples for analyses of metals, minerals, trace elements, and PAHs were freeze dried in accordance with the AOAC 930.15 method as previously described [47]. All composite samples were analyzed for total protein, total fat and fatty acid (FA) content including saturated fatty acids (SFA), sum monounsaturated fatty acids (MUFA), sum polyunsaturated fatty acids (PUFA), n-3/n-6 ratio, eicosapentaenoic acid (EPA), and DHA. Furthermore, the concentration of vitamins A (A_1_ and A_2_) and D_3_, calcium and trace elements iodine, selenium, zinc, and iron were analyzed. The concentration of heavy metals arsenic (total arsenic), mercury (total mercury), lead, and cadmium was analyzed in all composite samples in addition to levels of PAHs. All analyses were performed in singular parallels at the IMR laboratories using accredited methods with NS-EN ISO/IEC 17025 standards, except for iron. The analytical methods including corresponding limits of quantification (LOQ) and measurement uncertainties are described in detail elsewhere [48].

#### Determination of crude protein, crude fat and fatty acids

The crude protein content was determined by burning the material in a combustion tube containing pure oxygen at 830°C. Nitrogen (N) was detected with a thermal conductivity detector (TCD), and the content of N was calculated from an estimated average of 16% N per 100 g protein using the following formula

Ng/100g×6.25=proteing/100g.

The method is accredited according to AOAC Official Methods of Analysis [49].

For determination of crude fat, the fat content was extracted with ethyl acetate and filtered before the solvent was evaporated and the residual fat was weighed. The method is accredited in accordance with ISO-EN 17025 and standardized as a Norwegian Standard, NS 9402 [50].

The fatty acid content was determined using gas liquid chromatography (GLC). Lipids in the samples were extracted according to Folch, Lees [51], and the fatty acid composition of total lipids was analyzed as previously described in Fauske, Bernhard [52]. Methyl esters were separated on a capillary gas column (CP-sil-88, 50 m WCOT, ID:0.32) and peaks were identified by retention time using standard mixtures of methyl esters (Nu-Check, Eylian, USA). Content of fatty acids per gram sample was subsequently calculated using 19:0 methyl ester as an internal standard.

#### Determination of vitamins

To determine vitamin A_1_ (sum all trans-retinol and 13-, 11-, 9 cis retinol) and A_2_ (4,4 didehydro-all-trans retinol) content, the samples were saponified and the unsaponifiable material was extracted. Vitamin A_1_ and A_2_ was determined by high-performance liquid chromatography (HPLC) (normal phase) using a PDA detector (Photo Diode Array) and the retinol content was calculated by external calibration (standard curve) [53].

For determination of vitamin D_3_, the samples were saponified, and the unsaponifiable material was extracted and subsequently purified in a preparative HPLC column. The fractions containing D_2_ (ergocalciferol) and D_3_ (cholecalciferol) were pooled (normal phase) and injected on an analytic HPLC column (reverse phase). Vitamin D_3_ and D_2_ was determined by an ultraviolet (UV) detector and the content of vitamin D_3_ was calculated using vitamin D_2_ as an internal standard [54].

Vitamin B_12_ (Cobalamin) was released from the sample by extraction (autoclaving in acetate buffer) and mixed with growth medium before the microorganism (*Lactobacillus delbruecki* -ATCC 4797) was added and subsequently incubated at 37°C for 22 hours. The concentration of vitamin B12 was calculated by comparing the growth of the organism in the unknown samples with the growth of the organism in known standard concentrations by turbidimetric reading (Optical Density, OD, v / 575 nm) [55].

#### Determination of metals, minerals and trace elements

The concentration of calcium, heavy metals cadmium, arsenic (total), mercury (total), and lead as well as trace elements selenium, zinc, and iron was determined by Inductively Coupled Plasma-Mass Spectrometry (iCapQ ICP-MS, ThermoFisher Scientific, Waltham, MA, USA) equipped with an autosampler (FAST SC-4Q DX, Elemental Scientific, Omaha, NE, USA) after wet digestion in a microwave oven as previously described by Julshamn, Maage [56]. For determination of iodine, the sample was extracted with tetramethylammonium hydroxide (TMAH) before ICP-MS analysis. The elements were quantified using an external standard curve in addition to different internal standards for specific elements; scandium (Sc) was used as internal standard for calcium, rhodium (Rh) was used as internal standard for zinc and selenium, tellurium (Te) was used for iodine and either Rh, germanium (Ge), indium (In) or thulium (Tm) was used for the heavy metals.

#### Determination of PAHs

The concentration of the PAHs benz(a)anthracene (BaA), benzo(a)pyrene (BaP), benzo(b)fluoranthene (BbF) and Chrysene (Chr), collectively referred to as PAH4, were determined by Gas Chromatography Mass Spectrometry (GC-MS/MS) after extraction with dichloridemethane (DCM): cyclohexane (1:3) using an US EPA 16 PAH cocktail (CIL ES-4087) internal standard. The extract was evaporated and rinsed on an SPA column (Envichrom) before adding recovery standard and analyzing the samples by GC-MS/MS [57, 58].

### Microbial analysis

#### Sample preparation

After arrival at the German Federal Institute for Risk Assessment, the samples were stored at 4°C until further processing. Samples were homogenized batchwise using a GRINDOMIX GM200 (Retsch GmbH, Haan, Germany) for 20 seconds at 4.000 rpm and stored in plastic cups with a screw-on lid (WMC Medical Consulting, Pulheim, Germany) at 4°C until microbiological analysis.

#### Rehydration of fish and preparation of pooled initial suspension

The batchwise samples were processed as described in ISO 6887–3:2003. A batch sample aliquot of 11 g was mixed with 22 g buffered peptone water (Mast Group, Bootle, United Kingdom) in a peristaltic lab blender (Bag Mixer CC, Interscience, Saint Nom, France) for 10 seconds and rehydrated at room temperature for 1 h. The batchwise initial suspensions were prepared as a tenfold dilution of the rehydrated fish by addition of 297 g of buffered peptone water and mixing it for 90 seconds. For microbial analysis, initial suspensions of batch samples were pooled per species and market, as described for the analysis of nutrients and minerals.

#### Analysis of total colony count, *E*. *coli*, coliform bacteria and *Salmonella* spp.

The total colony count (TCC) analysis was based on ISO 4833:2015, however, instead of plate count agar, Columbia blood agar plates (Oxoid Deutschland GmbH, Wesel, Germany) were used. The pooled initial suspension was further diluted 1:10 in maximum recovery dilution (Oxoid Deutschland GmbH, Wesel, Germany) and 100 μL per dilution step were spread on agar plates. One set of plates were incubated at 30°C for 72 h aerobically, and an additional set of plates were incubated at 30°C for 72 h under anaerobic conditions in anaerobic jars with AnaeroGen^™^ sachets (Oxoid Deutschland GmbH, Wesel, Germany). The limit of detection (LOD) based on the detection of one colony per plate was calculated as 2.5 log CFU/g of processed fish.

For quantification of *E*. *coli* and coliform bacteria, Brilliance^™^ *E*. *coli*/coliform agar plates (CM1046, Oxoid Deutschland GmbH, Wesel, Germany) were inoculated with 100 μL of the pooled initial suspension and their further dilutions. In addition, 1 mL of the pooled initial suspension was spread on three plates to increase the level of detection 10-fold (LOD 1.5 log CFU/g). The plates were incubated at 37°C for 24 h, aerobically.

The detection of *Salmonella* spp. was performed according to ISO 6579–1:2017. The 300 mL initial suspensions per batch were incubated at 37°C for 18 h, aerobically. After incubation, enriched batch samples were pooled as described above. An aliquot of 1 mL of the pooled enrichment culture was added to 10 mL Mueller Kauffman Tetrathionate Novobiocin broth (MKTTn; Oxoid Deutschland GmbH, Wesel, Germany) and incubated at 37°C for 24 h, aerobically. Further, 100 μL was dripped as three droplets on modified semi-solid Rappaport Vassiliadis agar (MSRV; Oxoid Deutschland GmbH, Wesel, Germany) and incubated at 41.5°C for 24 h, aerobically. After incubation, 10 μL of MKTTn selective culture or a loop full of spreading growth from MSRV were spread to Xylose Lysine Deoxycholate agar plates (XLD, Merck, Darmstadt, Germany) and Gassner agar plates (GAS, sifin, Berlin, Germany) both incubated at 37°C for 24 h aerobically to achieve single colonies. After incubation, plates were checked for presumptive colonies.

### Data management

The analytical data was exported from Laboratory Information Management Systems (LIMS) and processed using Microsoft Excel 2013. When calculating mean and standard deviation, analyses below LOQ were included in the calculations as the respective LOQ value divided by two. Statistical analyses was performed using IBM SPSS version 26 (IBM Corp., Armonk, NY, USA) and for all tests a significance level of α = 0.05 was used. Normal distribution was tested using Shapiro-Wilk test. For normally distributed data, the homogeneity of variances was tested using Levene-test. If homogeneity of variances was given, the differences between the fish species was analyzed using one-way analysis of variance (ANOVA). As a post hoc test to discriminate different pairs, a Tukey-test was performed. If the homogeneity of variances was not given, a Welch-ANOVA was used to determine the differences between the fish species, followed by a Dunnet-T3-test. If the values were not normally distributed, a Kruskal-Wallis-test was performed to determine differences between the species followed by pairwise comparison of the fish species with a Bonferroni correction.

## Results and discussion

In the present study, analytical data on 24 composite samples of processed, small fish (total length 4.6–13.3 cm) from several fish markets covering a large geographic range in Ghana are reported (Table 1). Based on the nutritional analyses all the processed fish samples, except tilapia, had the potential to significantly contribute with essential nutrient to the intakes of vitamins, minerals, and essential fatty acids. In all samples, the microbiological total cell counts were acceptable (Table 2). High levels of PAHs, mercury and lead were detected in some samples (Table 2), which calls for further research and identification of anthropogenic sources in different regions and along the value chains.

**Table 2:** Analytical values (means ± standard deviation, wet product weight) for selected nutrients, microbial quality and contaminants of processed whole fish from Ghana.

		Unit	Anchovy	Bigeye grunt	Round sardinella	African moonfish	WA pygmy herring	Tilapia	p-value
**Moisture content**		%	8.45±1.31^AB^	8.22±1.77^AB^	7.92±1.75^A^	10.36±1.66^AB^	7.87±2.04^AB^	28.50±7.96^B^	0.019
**Nutrients**	**Protein**	g/100g	71.80±2.05^A^	64.33±1.53^B^	64.80±3.19^B^	67.67±1.53^AB^	67.67±3.06^AB^	31.80±3.90^C^	<0.001
	**Total Fat**	g/100g	6.44±0.77^A^	15.03±3.80^AB^	13.84±1.29^B^	6.37±0.97^A^	12.23±3.38^AB^	7.02±2.11^A^	0.001
	**Sum SFA**	g/100g (%)	2.00±0.47^A^ (37)	5.16±1.44^B^ (38)	4.94±0.77^B^ (33)	1.99±0.21^A^ (38)	4.02±0.78^BC^ (39)	2.93±0.38^AC^ (41)	<0.001
	**Sum MUFA**	g/100g (%)	0.75±0.13^A^ (14)	3.20±1.01^B^ (24)	2.48±0.52^AB^ (26)	0.92±0.10^AB^ (18)	2.86±0.97^B^ (27)	2.06±0.36^AB^ (28)	0.003
	**Sum PUFA**	g/100g (%)	2.33±0.58^AB^ (43)	4.25±1.04^C^ (32)	3.98±0.58^C^ (35)	1.92±0.20^AB^ (37)	3.12±0.63^AC^ (30)	1.68±0.17^B^ (23)	<0.001
	**EPA**	g/100g (%)	0.41±0.13^AB^ (7)	0.77±0.26^B^ (6)	0.82±0.17^B^ (7)	0.34±0.07^AB^ (6)	0.29±0.06^AB^ (3)	0.04±0.01^A^ (1)	0.001
	**DHA**	g/100g (%)	1.41±0.32^A^ (26)	2.21±0.47^B^ (16)	2.01±0.30^B^ (16)	1.04±0.12^AC^ (20)	0.77±0.22^CD^ (8)	0.18±0.06^D^ (3)	<0.001
	**Vitamin B**_**12**_	μg/100g	14±3^AB^	9±1^A^	23±1^B^	14±1^A^	16±6^AB^	11±3^A^	0.014
	**Vitamin D**_**3**_	μg/100g	12±3^AB^	15±6^AB^	34±9^A^	9±3^B^	17±10^AB^	13±4^AB^	0.024
	**Vitamin A**_**1**_	μg/100g	14±15^AB^	323±153^B^	10±3^AB^	290±46^B^	39±28^AB^	4±8^A^	0.004
	**Calcium**	mg/100g	2940±207^AB^	3533±577^ABC^	3040±207^B^	5467±153^C^	2633±379^AB^	2900±436^AB^	0.026
	**Iron**	mg/100g	25±6^A^	22±9^AB^	19±3^A^	50±12^AB^	78±89^AB^	10±3^B^	0.010
	**Zinc**	mg/100g	6.3±0.9^ABC^	3.4±0.4^BC^	5.1±0.2^ABC^	4.9±1.0^ABC^	14.7±2.9^A^	4.3±0.6^BC^	0.003
	**Selenium**	μg/100g	192±28^A^	113±12^B^	242±37^A^	173±15^A^	94±15^BC^	33±7^C^	<0.001
	**Iodine**	μg/100g	170±48^A^	219±122^A^	142±31^AB^	233±25^A^	129±74^AB^	49±11^B^	0.002
**Microbial Quality**	**TCC aerob**	log CFU/g	5.06±0.74^A^	4.58±0.23^A^	4.62±0.52^A^	4.93±0.30^A^	6.15±0.74^A^	4.23±0.64^A^	0.077
	**TCC anaerob**	log CFU/g	4.36±0.92^A^	3.67±0.42^A^	4.05±0.92^A^	3.98±0.27^A^	NA^A^	4.43±1.01^A^	0.505
	**Coliform**	log CFU/g	2.73±0.85^A^	2.86±1.05^A^	2.93±1.19^A^	2.69±0.07^A^	3.12±1.28^A^	<LOD^A^	0.639
**Contaminants**	**PAH4**	μg/kg	478±164^AB^	553±155^A^	418±103^AB^	443±91^AB^	600±666^AB^	7±10^B^	0.034
	**Cadmium**	mg/kg	0.306±0.053^A^	0.116±0.016^BC^	0.186±0.031^B^	0.065±0.019^CD^	0.015±0.004^D^	<LOQ^D^	<0.001
	**Lead**	mg/kg	0.13±0.06^A^	0.16±0.11^A^	0.10±0.04^A^	0.24±0.10^A^	0.64±0.61^A^	<LOQ^A^	0.059
	**Mercury**	mg/kg	0.034±0.006^AB^	0.065±0.014^A^	0.034±0.009^AB^	0.045±0.013^AB^	0.223±0.127^A^	<LOQ^B^	0.003
	**Arsenic**	mg/kg	7.8±1.9^A^	4.9±0.6^A^	9.8±3.0^A^	5.7±1.4^AB^	0.9±0.6^B^	0.1±0.0^B^	<0.001

Different superscript capital letters indicate statistical significance with p-value below 0.05. <LOD—Mean value below limit of detection; <LOQ—Mean value below limit of quantification; NA- Not available; SFA—saturated fatty acids; MUFA—monounsaturated fatty acids; PUFA—polyunsaturated fatty acids; EPA—eicosapentaenoic acid; DHA—docosahexaenoic acid; TCC—total colony count; PAH4 –sum of benz(a)anthracene, benzo(a)pyrene, benzo(b)fluoranthene and Chrysene.

### Nutrient content

#### Proximate and fatty acid composition

The content of protein and fat as well as fatty acid composition of the processed fish species are presented in Table 2 and in S3 Table. Protein content ranged from 31.80 g/100 g in salted tilapia to 71.80 g/100 g in smoked European anchovy, thus constituting a significant source of complete proteins in the otherwise starchy-staple-dominant Ghanaian diet. The stability of proteins in fish have previously been examined, and analyses of sardine (*Sardinella* spp.) from Ghana reported minimal variation in protein content between fresh and smoked specimens [60]. Furthermore, Usydus, Szlinder-Richert [61] found that in terms of both quality and quantity, the amino acids in smoked and salted fish remain stable and highly digestible according to WHOs protein standard [61]. Despite being subjected to a variety of processing techniques, dried, salted and smoked fish from Ghana can thus be regarded as a high-quality protein source [62].

Fat content in fish is more variable than other proximate components and may reflect a natural variance in different fish species along with seasonal variations in feed sources and availability for the given species [63]. A considerable range in fat content was thus expected and found (from 6.37 g/100 g in smoked African moonfish to 15.03 g/100 g in smoked bigeye grunt). A noteworthy variation in the fat content of WA pygmy herring was observed between the different markets (8.4–14.8 g/100 g), which consisted of both dried and smoked composite samples. It has been reported that smoking may have a modifying effect on the concentration of fat in fish [64, 65]. However, by taking the FA compositions into consideration, we theorize that the variance observed in WA pygmy herring may be attributed to the use of plant-based cooking oil during processing.

The marine products smoked bigeye grunt and smoked round sardinella had the highest mean values of the n-3 fatty acids EPA and DHA, while smoked European anchovy had the highest content of EPA and DHA in terms of percentage. Salted tilapia and dried/smoked WA pygmy herring had the lowest content of EPA and DHA, which was expected given their freshwater origin. However, the difference to most marine water species was only significant for DHA. Interestingly, African moonfish had a similar content of EPA and DHA to the freshwater species WA pygmy herring. Further, the freshwater species WA pygmy herring showed high levels of PUFAs being more comparable to the sampled marine species and significantly higher than the levels of the freshwater species tilapia. Fatty acids are highly susceptible to oxidation; however, previous studies have documented that automated smoking of Atlantic mackerel (*Scomber scombrus*) and smoking of sardines (*Sardinella* spp.) and tilapia (*Tilapia* spp.*)* in a Ghanaian chorkor oven did not produce significant changes in fatty acid composition [64, 66]. However, the specific effects of different processing methods on fatty acid composition may not be concretized in the current study and is in need of further exploration.

#### Vitamin composition

The analytical values for vitamins D, A_1_ and B_12_ in the different processed fish species are presented in Table 2 and S2A–S2F Table. Vitamin D-levels were determined, ranging from 9.0 μg/100 g in African moonfish to 34.2 μg/100 g in round sardinella. It has been documented that the reduced water content from smoking increases the concentration of several nutrients [65], but heating processes have also proven detrimental to vitamin D retention [67]. Yet, the highest concentration of vitamin D in the current study from smoked round sardinella (34.0 μg/100 g) is similar to vitamin D levels in raw summer herring (11.5 μg/100 g) from the north Atlantic [68] when the difference in water content is adjusted for. Fish is one of few foods which naturally contain vitamin D and can therefore help to prevent vitamin D deficiency [69]. Deficiency in Vitamin D can lead to maldevelopment in the skeletal system and is presumed to also have a negative influence on the cardiovascular system [69–72]. Despite being subjected to different processing techniques, the analyzed fish may be considered valuable dietary sources of vitamin D.

Vitamin A_1_ content ranged considerably between species, from mean levels below the limit of quantification in salted tilapia to 323 μg/100 g in smoked bigeye grunt. To our knowledge, the impact of smoking on vitamin A degradation in fish has not been studied yet, but it has been established that sunlight and heat reduces vitamin A activity in foods up to 90% [73]. Vitamin A_1_ content could not be linked to fat content in the current study, introducing exposure to sunlight and high temperatures during processing and storage at fish markets as possible variables. Furthermore, Roos, Leth [74] documented that certain small fish species are valuable sources of vitamin A, with up to 50% of vitamin A concentrated in the eyes or in the viscera. It is worth mentioning that the fish with the highest vitamin A_1_ content, bigeye grunt, was the only fish analyzed without head. Analyses of vitamin A_2_’s characteristics are still in its inception, but current findings show that the bioavailability of vitamin A_2_ is higher (119–127%) than for A_1_ [75] and that some freshwater species consumed whole are a good dietary source [76]. In the present study, quantifiable levels of vitamin A_2_ were only detected in smoked marine fish species bigeye grunt (8.9 μg/100 g), round sardinella (5.2 μg/100 g) and African moonfish (3.4 μg/100 g). The ratio of vitamin A_2_ to total vitamin A content was low for bigeye grunt and African moonfish but constituted a considerable share (> 30%) in round sardinella, thus indicating that some processed marine fish species are also a potential source of vitamin A_2_. Collectively, our results suggest that including smoked bigeye grunt or smoked African moonfish in the diet may contribute towards alleviating the persistent burden of vitamin A deficiency in Ghana [77].

All fish species contained considerable amounts of vitamin B_12_, ranging from 8.9 μg/100 g in processed bigeye grunt to 23.0 μg/100 g in round sardinella. Animal source foods are known as the major dietary source of vitamin B_12_ [78] and previous studies have documented that the concentration of vitamin B_12_ is up to three times higher in the viscera of fish compared to fillet [79]. This indicates that consuming small fish whole may be suitable, particularly for population groups with limited access to animal source foods. A study on round herring *(Etrumeus teres)* demonstrated that heating processes may decrease vitamin B_12_ levels by up to 62% [79], but the final concentration of vitamin B_12_ in smoked, dried or salted fish could nevertheless be higher than in fresh fish. Still, the cumulative effects of high temperatures during both processing and cooking on vitamin levels are yet to be thoroughly investigated.

#### Mineral composition

Of the analyzed processed fish species, tilapia was the least mineral dense, containing the lowest concentrations of selenium (33 μg/100 g), iodine (49 μg/100 g) and iron (10 mg/100 g) compared with all other analyzed species, and significantly lower levels of selenium and iodine than the marine species. Round sardinella had the highest levels of selenium (242 μg/100 g) while African moonfish contained significantly higher concentrations of calcium (5467 mg/100 g) and iodine (233 μg/100 g). The smallest fish analyzed, WA pygmy herring, had the highest concentrations of both iron (78 mg/100 g) and zinc (15 mg/100 g). Even though mean iron values of WA pygmy herring were not significantly higher than for other species (Table 2), high levels of iron were detected in the composite sample of dried WA pygmy herring from Accra (180 mg/100 g; S2E Table), which exceeds previous findings of iron in smoked-dried tilapia from Ghana twentyfold [64]. The high values of iron measured in the specific composite sample of WA pygmy herring from Accra may be linked to the Agbogbloshie area in Accra where the ambient metal contamination is high due to burning of e-waste. But further studies are needed to verify this potential regional contamination pathway. Still, with a high prevalence of iron deficiency anemia [80], the inclusion of processed small fish could be a valuable addition to the staple-dominant Ghanaian diet by providing bioavailable iron [3, 9, 81, 82].

Zinc is essential for optimal growth, and is closely interlinked with aggravated symptoms of iron deficiency anemia with its role as a catalyst in iron metabolism [83]. Although the prevalence of zinc deficiency has not yet been evaluated in Ghana, it can be assumed that a diet comprising limited animal-source foods is inherently low in terms of both zinc content and bioavailability [13]. Zinc levels in the current study were similar to previously determined levels in smoked sardine (S*ardinella* spp) from Ghana [64] and notably higher than the concentration in small raw freshwater fish analyzed whole [84]. These findings also correspond with the reported stability and high retention of zinc documented in cooked foods [85, 86]. Incorporating all processed fish species, but primarily WA pygmy herring, in the diet could thus be a valuable contribution towards increasing dietary zinc and simultaneously alleviating iron deficiency anemia in Ghana.

Adequate iodine intake is essential for synthesis of thyroid hormones and neurodevelopment, however, the magnitude of iodine deficiency among children and other vulnerable population groups in Ghana is not thoroughly mapped. Marine fish are regarded as the superior dietary source to iodine [87]. However, iodine content largely depends on environmental conditions, and large variations are expected both between and within different fish species [88, 89]. This great variation was confirmed in the present study, with iodine content in bigeye grunt ranging from 96–340 μg/100 g. As a freshwater fish, processed tilapia showed significantly lower iodine levels than most of the marine species. Interestingly, the freshwater species WA pygmy herring showed relatively high iodine levels which were comparable to the processed marine species. In line with our results, former analyses of iodine in whole smoked European sprat (*Sprattus sprattus*) have yielded high average levels of iodine (148 μg/100 g) and iodine retention ranged from 48–93% in a study on different cooking methods [90], thus underlining that processed and cooked fish may be considered a good source of dietary iodine.

Selenium plays a key role in ameliorating the toxic effects of mercury [91] which is present in variable concentrations in fish [92, 93]. Previous analyses of smoked European sprat (*Sprattus sprattus*) reported a selenium content of 20 μg/100 g [94], which is markedly lower than for all fish species analyzed in the current study. With the high levels of selenium determined in our study we conclude that along with fresh seafood [95], processed fish may be considered an excellent dietary source of selenium.

### Food safety

#### Microbiological quality

**Fig 1:**
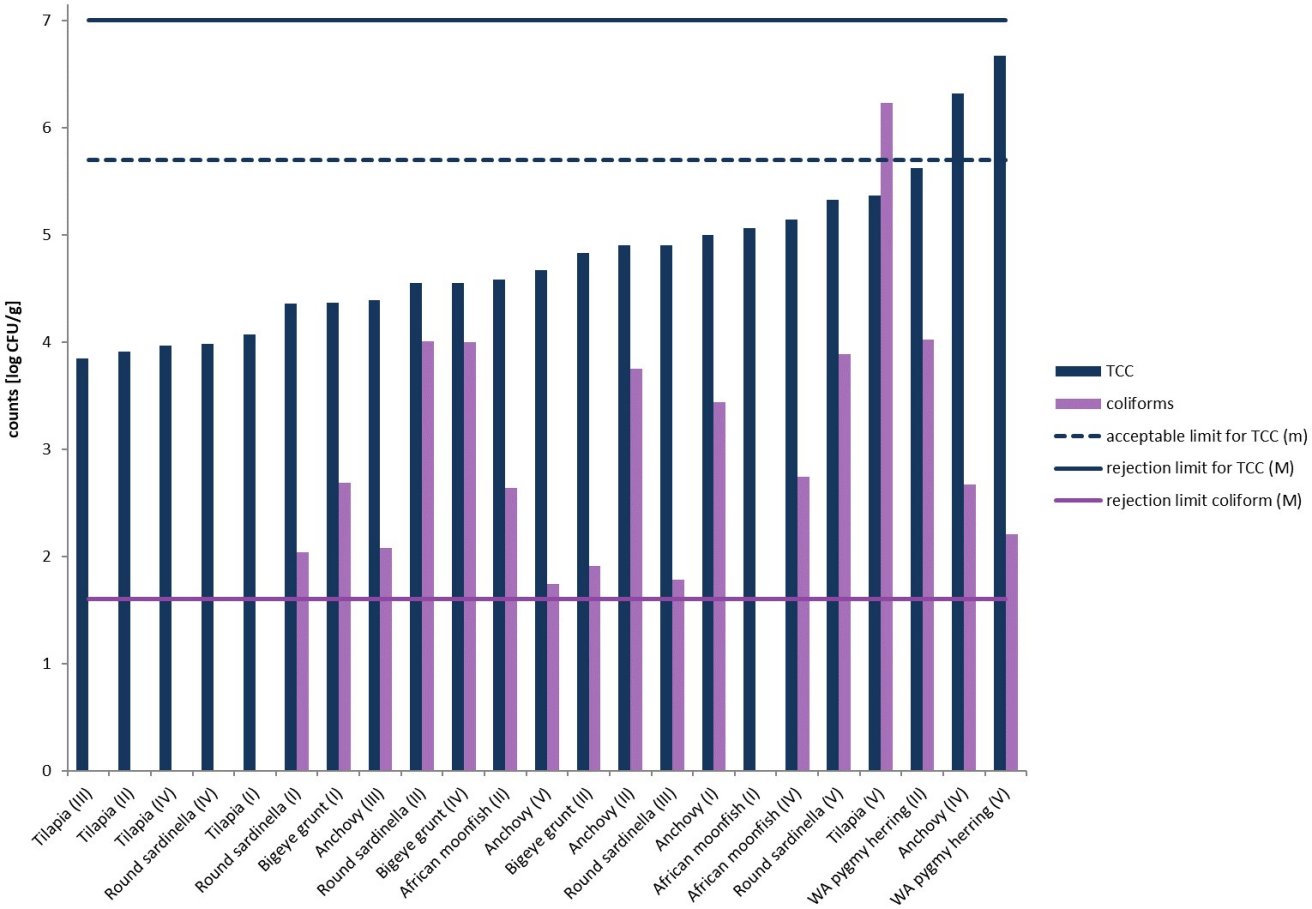
Total Colony Counts (TCC), counts of coliform bacteria (coliforms) and the acceptable limit (m, 5.7 log CFU/g) for TCC, the rejection limit for TCC (M, 7 log CFU/g) and the rejection limit for coliforms (M, 1.6 log CFU/g) by the Ghana Standards Authority (GS 95:2013). I: Accra; II: Techiman; III: Tamale; IV: Kumasi; V: Bolgatanga.

A total of 24 pooled samples were analyzed for bacteria related to microbiological quality. Total colony count (TCC) was used to describe the general bacterial load which can be used as an indicator for poor hygiene conditions and control along the value chain. Coliform bacteria and *E*. *coli* were used as indicator bacteria for fecal contamination along the value chain and *Salmonella* was analyzed as their presence is associated with food-borne disease. The microbial quality parameters of the processed fish species are presented in Fig 1 and Table 2.

**Fig 2:**
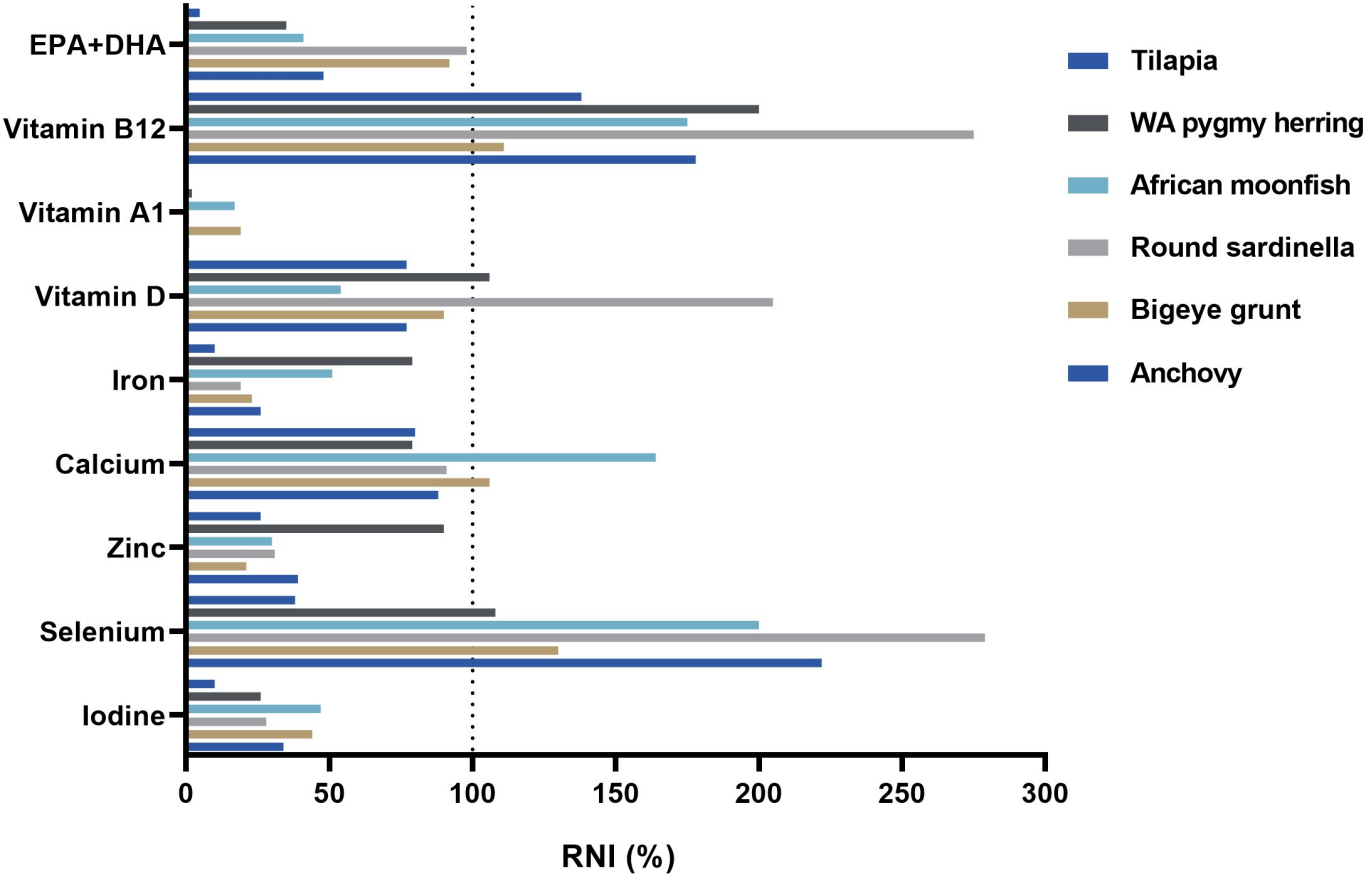


As shown in Fig 1, the highest aerobic TCC were found in WA pygmy herring from Bolgatanga (6.67 log CFU/g) while the lowest counts were detected in tilapia from Tamale (3.85 log CFU/g). The two sampled freshwater fish species differed in two orders of magnitude in mean aerobic TCC with 6.15±0.74 log CFU/g in WA pygmy herring and 4.23±0.64 log CFU/g in tilapia. While in marine species, mean aerobic TCC ranged between 5.06±0.74 log CFU/g (European anchovy) and 4.58±0.23 log CFU/g (bigeye grunt). The Ghana Standards Authority provides limits for aerobic TCC in hot smoked fish with an acceptable limit (m) for TCC at 5.7 log CFU/g and a rejection limit (M) for TCC at 7 log CFU/g (GS 95:2013). None of the here analyzed samples had an aerobic TCC above the rejection limit (Fig 2), but European anchovy samples from Kumasi (6.32 log CFU/g) and WA pygmy herring from Bolgatanga (6.67 log CFU/g) had TCCs above the acceptable limit. This means that more than 90% of all samples were within the Ghanaian acceptable limit for aerobic TCC. There are no scientific reports available on aerobic TCC in processed fish from Ghanaian markets and only few reports are available from other African countries. In Nigeria, Adegunwa, Adebowale [96] analyzed smoked herring (*Sardinella eba*) samples from three different markets and found aerobic TCC ranging from 6.36 log CFU/g to 7.47 log CFU/g, thus being more than one log-cycle higher than in the here presented samples in general. Another study from Nigeria by Akinwumi and Adegbehingbe [97] reported mean aerobic TCC ranging between 4.11 log CFU/g and 5.46 log CFU/g in freshwater fish species (tilapia and African mud catfish) and 4.70 log CFU/g and 5.46 log CFU/g in herring from three different markets. In smoked catfish from Malawi, high aerobic TCC (6.75 log CFU/g) were found by Likongwe, Kasapila [24], who compared two different hot smoking techniques. In view of the few published market studies from other African countries, the here analyzed samples of Ghanaian processed fish showed lower aerobic TCC mainly within the acceptable limit. However, aerobic TCC can only be used as an indicator for general hygiene conditions along the value chain and should be combined with more specific microbial identification.

In the sampled fish products, anaerobic TCC were showing low levels being one order of magnitude below the levels of aerobic TCC (Table 2). Consequently, the microbiota on these processed fish products were dominated by aerobic and facultative anaerobic organisms. For anaerobic TCC, no Ghanaian reference values are available. The presence of anaerobic growing bacteria was low in the analyzed samples, but to exclude the presence of hygienically relevant bacteria, further identification is needed.

The analysis of *E*. *coli* in sampled processed fish from Ghana revealed that only one composite sample (WA pygmy herring from Techiman) was positive for *E*. *coli* with a concentration of 2.51 log CFU/g). Higher levels were found for coliforms in 70.8% of the fish samples. There was a strong variance in contamination of single fish species with coliforms between market samples, where the highest coliform counts were found in tilapia from Bolgatanga (6.23 log CFU/g, Fig 1) while tilapia samples from other markets were negative for coliform bacteria, which is calling for identification of sources within the affected value chains. The mean concentrations of coliform bacteria in European anchovy, bigeye grunt, and African moonfish were 2.73±0.85 log CFU/g, 2.86±1.05 log CFU/g, and 2.69±0.07 log CFU/g, respectively. European anchovy, bigeye grunt, WA pygmy herring tested positive for coliforms in all samples whereas round sardinella from two markets (Tamale and Kumasi) and African moonfish from Accra tested negative for coliforms. The guidelines by the Ghana Standards Authority set the limit for coliform bacteria in hot smoked fish to 1.6 log CFU/g (GS 95:2013). Based on the LOD in the present study, all samples that tested positive for coliform bacteria thus exceeded the legal limit in Ghana. The high concentrations of coliform bacteria in most of the samples might indicate fecal origin, therefore contamination from a dusty environment, hygienic conditions, contact surfaces, or handling practices are likely sources. The large variations in coliform counts, both between different products and between markets, indicate the potential to mitigate the bacterial load by improving hygienic conditions along the value chains. Critical points along the value chains might differ locally, which necessitates deeper investigation in future studies.

*Salmonella* was analyzed in this study as a food-borne pathogen, but was not detected in any of the sampled processed fish. Similar to our results, Plahar, Nerquaye-Tetteh [29] did not detect the presence of *Salmonella* in two different processed marine species in Ghana and Adegunwa, Adebowale [96] did not detect *Salmonella* in smoked herring samples from three different markets in Nigeria. In contrast, Likongwe, Kasapila [24] analyzed smoked catfish from Malawi and detected *Salmonella* with counts up to 4 log CFU/g in fresh and smoked fish. Further, *Salmonella/Shigella* was found in catfish, tilapia, and herrings collected from different markets in Nigeria [97]. Based on our results in combination with published data, *Salmonella* was generally not present in processed fish samples, but might be a possible food-borne hazard in certain fish value chains.

The sampling approach in this study was endpoint oriented and showed that aerobic TCCs were in the acceptable range, while only one sample tested positive for *E*. *coli* and no *Salmonella* were detected. But the counts of coliform bacteria were exceeding the limit given by the Ghana Standards Authority (Fig 1) in the majority of the samples and might indicate fecal contamination. As coliform bacteria and *E*. *coli* are foremost fecal bacteria and are classified as indicator bacteria for fecal contamination of food that harbors an increased risk to contain pathogenic bacteria [97, 98], several points along the value chains are possible critical points where contamination could have taken place. Either, the fish was primarily populated from its habitat and the smoking and drying of the fish was not sufficient to reduce coliform counts, or the smoked products were re-contaminated along the value chain due to improper handling or storage. Distinct conclusions on the effect of different preservation techniques (e.g. smoking, salting, and drying) or the different habitats cannot be drawn from this study design and need further research.

To develop suitable, locally applicable strategies to minimize the contamination by coliforms, critical points along the specific value chains need to be identified in further studies. Such strategies might include training in best practice procedures and process control to improve hygienic conditions, processing techniques, storage conditions and transportation. At consumer level, it is recommended to thoroughly cook fish before consumption to minimize the risk of food-borne illnesses.

#### Polycyclic aromatic hydrocarbons (PAHs)

Different PAHs are known to have carcinogenic and mutagenic properties [23, 25, 26]. To determine the amount of the most important PAHs in food, the European Commission recommends an analysis for benzo[a]anthracene, chrysene, benzo[b]fluoranthene, and benzo[a]pyrene, summarized as PAH4 [25, 99, 100]. All processed marine fish products analyzed in this study were smoked. Processed European anchovy, bigeye grunt, round sardinella, and African moonfish showed high mean PAH4 concentrations of 478±164 μg/kg, 553±155 μg/kg, 418±103 μg/kg, and 443±91 μg/kg, respectively (Table 2). The freshwater fish in this study were either smoked (WA pygmy herring from Accra and Bolgatanga), dried (WA pygmy herring from Techiman), or salted (all tilapia samples) (Table 1). The PAH4 concentration of WA pygmy herring differed between the pooled market samples, probably because the pooled samples were differently composed of smoked and not-smoked batches. In salted tilapia, the mean PAH4 level (7.4±9.6 μg/kg) was lower than all composition samples of smoked fish species (Table 1). There are no legal limits available from the Ghana Standards Authority for PAHs in fish products and the European limit value of 12.0 μg PAH4/kg of the product was therefore used as reference [101]. PAH4 levels exceeded the EU limit considerably in all market samples of processed European anchovy, bigeye grunt, round sardinella, and African moonfish as well as in WA pygmy herring from the markets in Techiman and Bolgatanga (Table 2). Only one composite sample of smoked fish did not exceed the EU limit for PAH4 (smoked WA pygmy herring from Accra, S2E Table). Generally, salted tilapia samples were below the EU limit value (except the samples from Bolgatanga containing 24 μg PAH4/kg, S2F Table). PAHs are known to be produced during the smoking process, and therefore levels in non-smoked fish species are expected to be low. Based on the literature, levels of PAH4 are highly influenced by the type of smoking kiln, type of burning material, duration of smoking, re-smoking of fish products and fat content of the fish [23, 26, 102, 103]. Consequently, the PAH4 levels in fish products can be minimized by applying best practices within the smoking process. Given the high concentration of PAH4 in the majority of smoked fish samples in this study, applied smoking techniques and smoking practices in Ghana should generally be improved. The United Nations (UN) in cooperation with the Food and Agriculture Organization (FAO) developed an improved kiln called the FAO-Thiaroye Processing Technique (FTT) [23, 26]. In this improved kiln, fat and other fluids from the fish are not able to drip into the fire. Further, the fish are not in direct contact with flames and the smoking process can be performed in a more controlled way, achieving up to a 100-fold reduction in PAH4 concentration in smoked fish when using FTT compared to traditional smoking methods [23, 26]. However, the FTT is more expensive, and the Ahotor oven [104–106] was therefore developed in cooperation with the United States Agency for International Development (USAID) as a more affordable option. This oven is equipped with a fat collector, requires less burning material compared to the chorkor and is assumed to produce less PAHs. The awareness of the negative effect of PAHs on human health should be widely communicated to the public and the use of safer ovens such as the FTT and the Ahotor oven and best smoking practices should be advocated to limit the health hazard for the consumer to a minimum.

#### Heavy metals

Heavy metals can have neurodegenerative as well as nephro-, and immunotoxic effects on humans and are important contaminants regarding food safety. Fish are known to be a source of heavy metals, given their ability to bioaccumulate heavy metals from the surrounding water and feed.

Maximum levels for fish as food are available from Ghanaian regulations, the Codex Alimentarius Commission and the European Commission. As all these regulatory limits are given only for fresh fish or fresh fillet with a high moisture content, an application of these limits to processed/preserved fish products with lower moisture content requires a normalization of the analytical levels to a moisture content comparable with fresh fish [107]. EU legislation has established maximum permissible levels for cadmium (0.05 mg/kg wet weight (w.w.)), mercury (0.5 mg/kg w.w.), and lead (0.3 mg/kg w.w.), however, no value is available for arsenic [107]. The maximum level for lead in raw fish is in line with the guidelines provided by the Codex Alimentarius [108] but for arsenic compounds in fish, no maximum concentration has yet been established.

The concentration of cadmium varied from unquantifiable levels (<LOQ) in tilapia to 0.31 mg/kg in processed European anchovy (Table 2), which was significantly higher than for all other species analyzed. Overall, the cadmium concentrations were significantly lower in freshwater species compared with marine species, with the exception of African moonfish. The Ghanaian regulation does not give limit values for processed fish and aligns with the limit values set by the European Commission for cadmium concentration in raw fish at 0.05 mg/kg wet weight and 0.25 mg/kg wet weight for *Engraulis species* [107, 109]; thus, a reduction in water content due to processing needs to be taken into account [109]. When normalizing the analytical levels to a 75% moisture content as in fresh fish [110–113], the concentration of heavy metals in the sampled fish species were below their respective limit values given by the European Commission. After normalizing to a 75% moisture level, the cadmium levels in marine species ranged from approximately 0.009 mg/kg in African moonfish to 0.034 mg/kg in European anchovy. Kwaansa-Ansah, Nti [114] reported cadmium concentrations in marine fish fillets from Ghana ranging from 0.007 to 0.019 mg/kg, which are in accordance with the findings in this study, with only anchovy having higher concentrations. Kortei, Heymann [115] reported cadmium concentrations in fillet of two freshwater species from below detection limit up to 0.08 mg/kg, which is higher compared to the samples analyzed in this study. Analytical data on heavy metal content including cadmium concentration in whole small fish for human consumption is scarce. Previous analyses have shown that cadmium primarily accumulates in the gills, liver, and kidneys and therefore fish species consumed whole might contain higher concentrations compared with consuming fillets [116, 117]. For cadmium, the EFSA recommends a Tolerable Weekly Intake of 2.5 μg/kg body weight [118], which would be reached by weekly consumption of 570 g of processed anchovy for a 70 kg person. However, risk assessment cannot be completed given the limited availability of food consumption data, data on smoked fish analyzed whole and overall exposure to cadmium. Further, regional differences reported in the present study should be further investigated in future studies.

Lead content in the analyzed processed fish ranged from levels below quantification in tilapia (<LOQ) to 0.64 mg/kg in WA pygmy herring. No statistically significant difference was detected between the different fish species, and consequently, no difference was detected between marine or freshwater habitats. However, WA pygmy herring from Accra were contaminated with lead at 1.3 mg/kg (S2E Table) and also had a high iron-content (see chapter on minerals). The high concentration in one species from one specific market might be caused by metal pollution from the Agbogbloshie e-waste recycling site close by, where lead is reported as the main toxicant [37, 119]. Nonetheless, it is unknown why only WA pygmy herring was affected, given that other species from this market did not contain notably higher levels and that WA pygmy herring from other markets did not show high levels of contamination. Consequently, future studies need to investigate sources of contamination including regional difference. No scientific papers analyzing the lead content of whole fish in Ghana were found for direct comparison, but studies of lead in muscle tissue of several freshwater species ranged from 0.04 to 0.42 mg/kg [115], 0.060–0.085 mg/kg in fillet of marine species [114] and levels lower than 0.2 mg/kg in fillet of catfish and bigeye grunt [120]. The lead concentration in dried fish products from our study was higher than levels reported for fresh fish, which is mainly caused by the reduced water content in the fish due to processing [121]. It is not yet known whether further contamination by lead takes place during processing. Nonetheless, with a concentration of 0.64 mg/kg processed fish, WA pygmy herring showed highest lead content within the sampled species, but after normalizing for water content it did not exceed the limit of 0.3 mg/kg flesh given by the European Commission [107]. However, given the overall high consumption of fish in Ghana and the neurodegenerative properties of lead, consumption pattern and exposure should be further investigated, especially in vulnerable population groups. The mechanism of lead absorption is similar to that of iron, and a diet deficient in iron can thus result in excess absorption of lead, particularly in young children [122, 123]. Given the high prevalence of iron deficiency among Ghanaian children and women of childbearing age [80], further analyses and monitoring of lead-levels in processed fish is essential including regional differences.

The mean concentration of mercury in most processed small fish was low, ranging from below quantification levels (<LOQ) in processed tilapia to 0.065 mg/kg in processed bigeye grunt. WA pygmy herring was an exception with a comparably high concentration of 0.223 mg/kg in all market samples (see S2E Table). It has been reported that freshwater species might contain higher concentrations of mercury compared to marine species [124]; however, this was not observed in this study, as the second freshwater species (tilapia) had the lowest concentration of all species. Total mercury concentration in fish depends on age, size and feeding habits combined with the general mercury concentration in the habitat, with the highest concentrations of mercury usually found in large predatory fish at peak trophic levels [125, 126]. The comparably high levels of mercury in WA pygmy herring thus contradict this rationale given its small size, short lifespan and herbivorous feeding habits. Data on mercury concentration in whole small fish as food for humans is scarce in the scientific literature, which limits the basis for comparison. However, scientific reports on mercury concentrations in fish fillet from the Gulf of Guinea and Ghana have reported mercury concentrations of 0.19 mg/kg up to 0.61 mg/kg in fillet of local marine and freshwater fish [34, 115, 120, 127]. The aforementioned studies found higher concentrations of mercury than in the present study, even though the water content was reduced by processing. Due to illegal gold mining, freshwater fish from surrounding watersheds are likely to be contaminated by mercury at higher levels [115, 127]. All samples analyzed in this study were below the limit given by the Ghanaian Standards Authority and European Commission of 0.5 mg/kg [107, 109]. For methylmercury, the JECFA set the PTWI to 1.6 μg/kg body weight [128]. Assuming all mercury in the samples is methylmercury, a 70 kg person would reach the PTWI by consuming 500 g of WA pygmy herring, which was the fish species with the highest concentration of mercury in this study. In marine fish, the highest concentration was found in bigeye grunt (0.065 mg/kg) and consumption of 1.7 kg of processed bigeye grunt would reach the PTWI, without taking any other sources of exposure into account. With regard to human health, fetuses, infants and children in early life stages are particularly susceptible to the neuro-, nephro-, and immunotoxic health hazards associated with mercury exposure [129], which emphasizes the importance of further studies on mercury in processed fish from Ghana including regional differences.

The concentration of total arsenic in all processed fish species had the highest variation in metal concentration with means ranging from 0.1 mg/kg in tilapia to 9.8 mg/kg in round sardinella. The sampled freshwater species, WA pygmy herring and tilapia, had significantly lower concentrations of arsenic compared with all marine species except African moonfish. To our knowledge, arsenic concentrations in whole fish have not been reported for Ghana thus far. Arsenic levels of 0.37 mg/kg have been reported in fillet of bigeye grunt, however, the samples were collected from only one landing site [120]. The current samples of whole processed bigeye grunt contained higher concentrations of arsenic in fish from all markets (S2B Table) and the other processed marine species showed similarly high concentrations of arsenic. In freshwater, Gbogbo, Arthur-Yartel [120] sampled Bagrid catfish (*Chrysichthys nigrodigitatus*) at one landing site and found arsenic concentrations of 0.21 mg/kg in the fillet. The WA pygmy herring analyzed in the current study displayed similar arsenic concentrations (after normalizing to 75% moisture content), while the sampled whole tilapia contained markedly lower arsenic levels. The low levels of arsenic in processed whole tilapia could not be explained with the current study-design, as it is unknown whether the tilapia were harvested from a freshwater system or if they originated from aquaculture. In this study, only total arsenic was analyzed, but it can be assumed that the less toxic organic arsenic is the dominant compound [130]. In Ghana and the EU no maximum limit for arsenic is provided for fish as food, and WHO/JECFA withdrew the PTWI for arsenic in 2011 [131]. The exposure to total arsenic for the Ghanaian population through consumption of processed whole fish is unknown, and the resulting health effects cannot be estimated without including local consumption data, which is currently missing.

Nti [10] have reported that 95% of households in rural Ghana consume fish on a daily basis. In combination with the challenges resulting from expanding anthropogenic activities, it is important to gain more knowledge on consumption habits to estimate the public risk resulting from heavy metal contamination in the different fish species including regional differences and other food commodities. As the limit values by the European Commission are only available for fresh fish and fillets, the change in concentration due to drying is an important factor to be considered for further risk assessment. For fish which are consumed whole, there is a need for additional food composition data on nutritional quality and contamination levels, given that there is a plausible difference between the consumption of whole fish and their fillets.

### Strengths and limitations of the study

The results provide novel data from Ghana on food quality and food safety including nutrient composition, heavy metal concentration, polycyclic aromatic hydrocarbon content and microbiological hygienic indicators in processed, i.e. smoked or dried whole small fish. As small fish are often consumed whole, the here presented data are highly valuable for future risk benefit analyses, given that existing data on fish is mostly limited to the content in the fillets of fresh fish. Still, some limitations are recognized. In the present study only pooled samples were analyzed, which would to some degree mask the presence of variance between the separate batches from the same sampling location. Further, the market sampling did not enable to trace the source and location of contamination as it was not known where and when the single fish batches were harvested, processed and stored. However, the advantage of this approach is that it gives a cross sectional outline on the nutrient composition, microbial concentration, and contamination with heavy metals and PAHs in Ghana. Origin and source distribution of the different contaminants, especially heavy metals, in the processed fish are relevant topics for future research. Especially heavy metal concentration is influenced by geographical origin of the unprocessed fish, but it might also be influenced by the location of processing, storage and marketing. Another aspect is the nutrient composition of processed fish which can vary and is affected by a multitude of pre-harvest and post-harvest factors. Pre-harvest factors include species, habitat, feed resources, life stage, seasonality, and changes in climatic patterns, while post-harvest factors include processing methods, storage conditions and shelf-life. Since this study gives an insight into the differences of the sampled processed fish, it is necessary to further investigate the aforementioned factors to display differences in nutrient composition and food safety issues throughout the year including different regions. Future research would need to take bioaccessibility of contaminants into consideration. Based on the here presented data, conclusions on the critical points along the value chains and geographical origin of the different contaminants cannot be drawn directly and need further investigation.

## Conclusion

Processed fish eaten whole are rich in vitamins, minerals and essential fatty acids and can therefore contribute with high-quality proteins and essential micronutrients in order achieve a balanced Ghanaian diet, which consists of mostly starchy staples. Of the analyzed fish species, tilapia stood out as markedly less nutrient dense. As tilapia is one of the species that could increase availability of fish through farming, the present results demonstrate that tilapia cannot substitute wild species in terms of micronutrient content. On the other side, the freshwater fish WA pygmy herring showed several nutritional characteristics of marine fishes. High concentrations of mercury and lead were detected in some fish samples, while elevated levels of PAHs were detected in all smoked samples. Heavy metal concentrations determined in this study call for further analyses and identification of geographical origin and contamination sources along the value chains to mitigate the health hazards associated with heavy metals. The high levels of PAHs in smoked fish in all regions necessitate the improvement of the smoking process by implementing best practices and improved kilns (e.g. FTT-kiln and Ahonto oven). Based on microbiological analysis, the overall quality of processed fish samples was acceptable. Nonetheless, the majority of sampled processed fish showed elevated coliform counts, but no *Salmonella* was found. The cause of contamination with coliform bacteria cannot be determined by this study and should be further investigated. Data from this study contributes to building reliable food composition data for Ghana on processed small fish. Further research is needed to analyze composition of prepared meals including preparation methods of small fish on consumer level. Having local consumption data is necessary to perform any risk benefit analysis and risk assessments and should be advocated in future studies.

## Supporting information

S1 Table (PDF)

S2 Table (PDF)

S3 Table (XLSX)
