# Supplementary material for: Composition of nutrients, heavy metals, polycyclic aromatic hydrocarbons and microbiological quality in processed small indigenous fish species from Ghana: Implications for food security
Source: PLoS One. 2020 Nov 12;15(11):e0242086. doi: 10.1371/journal.pone.0242086 (PMC7660496; doi:10.1371/journal.pone.0242086)
Supplement: S1 Table — (PDF) [file pone.0242086.s001.pdf]

**Analytical method**	**LOQ**	**Measurement uncertainly (%)**
**Total fat**	0,1 g/100 g	12 (0,1-5 g/100g)<br>8 (5-15 g/100g)
**Total Protein**	0,1 g nitrogen/100 g	40 (0,1-0,7g N/100g)<br>12 (0,7-16 g N/100g)
**Fatty acids**	0,1 %<br>0,001 g/100 g	100 (0,1 %)<br>50 (0,2-0,5 %)<br>10 (0,6-100 %)
**Vitamin D3**	1 µg/100 g	20
**Vitamin A**	0.3 µg/100 g	20
**Vitamin B12**	0.1 µg/100 g	30
**Minerals[a]**	Se 1 µg/100 g	25 % (40 % ≤LOQx10)
**(Se, Fe, Zn, Ca)**	Fe 0.01 mg/100 g<br>Zn 0.05 mg/100 g<br>Ca 3.5 mg/100 g	25 % (40 % ≤LOQx10)<br>20 % (40 % ≤LOQx10)<br>15 %
**Iodine[a]**	4 µg/100 g	40
**Elements[a]**<br>**(Hg, Pb, As, Cd)**	Hg 0.005 mg/kg<br><br><br>Pb 0.03 mg/kg<br>As 0.01 mg/kg<br>Cd 0.005 mg/kg	70 (0.005-0.05 mg/kg)<br>25 (0.05-0.5 mg/kg)<br>20 (0.5-4.6 mg/kg)<br>25 % (40 % ≤LOQx10)<br>20 % (40 % ≤LOQx10)<br>20 % (40 % ≤LOQx10)
**PAH4**<br>**(BaA, BaP, BbF, Chr)**	0.15 ng/g	15 %

[a]LOQ by dry weight
