# Supplementary material for: Composition of nutrients, heavy metals, polycyclic aromatic hydrocarbons and microbiological quality in processed small indigenous fish species from Ghana: Implications for food security
Source: PLoS One. 2020 Nov 12;15(11):e0242086. doi: 10.1371/journal.pone.0242086 (PMC7660496; doi:10.1371/journal.pone.0242086)
Supplement: S2 Table — (PDF) [file pone.0242086.s002.pdf]

S2A Table: Analytical values for selected nutrients, microbial quality and contaminant concentration in composite samples (n=5) of whole smoked European anchovy (including head, skin, viscera and bones) from selected cities in Ghana (mean±SD) [a]

		Unit	Accra[b]	Techiman[b]	Tamale[b]	Kumasi[b]	Bolgatanga[b]	Mean all cities
Nutrients	Protein	g/100g	70	70	74	71	74	72±2
	Total Fat	g/100g	7.2	6.1	6.3	7.2	5.4	6.4±0.7
	Sum SFA	g/100g (%)	2.0 (38)	1.8 (37)	1.9 (38)	2.8 (37)	1.6 (37)	2.0±0.5 (37)
	Sum MUFA	g/100g (%)	0.62 (12)	0.80 (16)	0.65 (13)	0.93 (12)	0.73 (17)	0.75±0.13 (14)
	Sum PUFA	g/100g (%)	2.4 (45)	2.1 (43)	2.2 (44)	3.3 (44)	1.7 (41)	2.3±0.6 (43)
	EPA	g/100g (%)	0.38 (7)	0.36 (7)	0.34 (7)	0.64 (8)	0.33 (8)	0.41±0.10 (7)
	DHA	g/100g (%)	1.4 (27)	1.3 (26)	1.4 (27)	1.9 (25)	1.0 (25)	1.4±0.3 (26)
	Vitamin B12	µg/100g	17	15	15	16	8.2	14±4
	Vitamin D_3	µg/100g	16	11	11	16	10	12±3
	Vitamin A_1	µg/100g	28	2.9	32	3.2	2.7	14±15
	Calcium	mg/100g	3100	3100	2900	3000	2600	2900±200
	Iron	mg/100g	22	30	17	25	32	25±6
	Zinc	mg/100g	7.2	6.7	6.3	6.6	4.8	6.3±0.9
	Selenium	µg/100g	220	160	220	190	170	190±28
	Iodine	µg/100g	180	140	150	250	130	170±48
Microbial Quality	TCC aerob	log CFU/g	5.00	4.90	4.39	6.32	4.67	5.06±0.74
	TCC anaerob	log CFU/g	3.91	4.23	3.39	5.85	4.44	4.36±0.92
	Coliform	log CFU/g	3.44	3.75	2.08	2.67	1.74	2.73±0.85
Contaminants	PAH4	µg/kg	510	360	410	750	360	478±164
	Cadmium	mg/kg	0.26	0.39	0.31	0.31	0.26	0.31±0.05
	Lead	mg/kg	0.24	0.12	0.078	0.093	0.12	0.13±0.06
	Mercury	mg/kg	0.028	0.042	0.038	0.035	0.027	0.034±0.006
	Arsenic	mg/kg	7.8	10	8.2	8.3	4.7	7.8±1.9

[a]Values are given as mean ± SD. [b] One composite sample was analyzed comprising >250 specimens in total. SFA - saturated fatty acids; MUFA - monounsaturated fatty acids; PUFA - polyunsaturated fatty acids; EPA - eicosapentaenoic acid; DHA - docosahexaenoic acid; TCC – total colony count; PAH4 – sum of benz(a)anthracene, benzo(a)pyrene, benzo(b)fluoranthene and Chrysene.

S2B Table: Analytical values for selected nutrients, microbial quality and contaminant concentration in composite samples (n=3) of smoked bigeye grunt (including skin, viscera and bones) from selected cities in Ghana (mean±SD) [a]

		Unit	Accra[b]	Techiman[c]	Kumasi[d]	Mean all markets
Nutrients	Protein	g/100g	63	64	66	64±2
	Total Fat	g/100g	19	13	13	15±4
	Sum SFA	g/100g (%)	6.6 (38)	3.7 (38)	5.2 (39)	5.2±1.4 (38)
	Sum MUFA	g/100g (%)	4.3 (25)	2.3 (23)	3.0 (23)	3.2±1.0 (24)
	Sum PUFA	g/100g (%)	5.3 (31)	3.2 (33)	4.3 (33)	4.3±1.0 (32)
	EPA	g/100g (%)	1.0 (6)	0.51 (5)	0.78 (6)	0.77±0.26 (6)
	DHA	g/100g (%)	2.6 (15)	1.7 (17)	2.3 (17)	2.2±0.5 (16)
	Vitamin B12	µg/100g	9.7	8.1	8.9	8.9±0.8
	Vitamin D_3	µg/100g	10	22	13	15±6
	Vitamin A_1	µg/100g	290	190	490	320±150
	Calcium	mg/100g	3200	4200	3200	3500±580
	Iron	mg/100g	32	20	15	22±9
	Zinc	mg/100g	3.2	3.9	3.1	3.4±0.4
	Selenium	µg/100g	100	120	120	110±12
	Iodine	µg/100g	340	96	220	220±120
Microbial Quality	TCC aerob	log CFU/g	4.37	4.83	4.55	4.58±0.23
	TCC anaerob	log CFU/g	3.38	3.97	NA	3.68±0.42
	Coliform	log CFU/g	2.69	1.91	4.00	2.86±1.05
Contaminants	PAH4	µg/kg	710	400	550	553±155
	Cadmium	mg/kg	0.13	0.12	0.098	0.12±0.02
	Lead	mg/kg	0.050	0.16	0.27	0.16±0.11
	Mercury	mg/kg	0.048	0.072	0.074	0.065±0.014
	Arsenic	mg/kg	4.3	5.0	5.4	4.9±0.6

[a]Values are given as mean ± SD. [b] One composite sample was analyzed comprising 62 specimens. [c] One composite sample was analyzed comprising 43 specimens [d] One composite sample was analyzed comprising 42 specimens. NA: Not available. SFA - saturated fatty acids; MUFA - monounsaturated fatty acids; PUFA - polyunsaturated fatty acids; EPA - eicosapentaenoic acid; DHA - docosahexaenoic acid; TCC – total colony count; PAH4 – sum of benz(a)anthracene, benzo(a)pyrene, benzo(b)fluoranthene and Chrysene.

S2C Table: Analytical values for selected nutrients, microbial quality and contaminant concentration in composite samples (n=5) of smoked round sardinella (including head, skin, viscera and bones) from selected cities in Ghana (mean±SD) [a]

		Unit	Accra[b]	Techiman[c]	Tamale[d]	Kumasi[e]	Bolgatanga[f]	Mean all markets
**Nutrients**	**Protein**	g/100g	69	63	64	61	67	65±3
	**Total Fat**	g/100g	12	13	14	15	15	14±1
	**Sum SFA**	g/100g (%)	5.8 (36)	4.2 (32)	4.1 (32)	5.5 (36)	5.2 (31)	4.9±0.8 (33)
	**Sum MUFA**	g/100g (%)	2.8 (25)	1.8 (28)	2.0 (25)	2.9 (22)	2.8 (32)	2.5±0.5 (26)
	**Sum PUFA**	g/100g (%)	4.0 (34)	3.9 (34)	3.1 (38)	4.6 (36)	4.3 (32)	4.0±0.6 (35)
	**EPA**	g/100g (%)	0.90 (8)	0.64 (7)	0.62 (6)	0.92 (6)	1.0 (8)	0.82±0.17 (7)
	**DHA**	g/100g (%)	1.9 (15)	2.2 (17)	1.6 (18)	2.4 (18)	2.1 (13)	2.0±0.3 (16)
	**Vitamin B12**	µg/100g	22	22	22	24	24	23±1
	**Vitamin D_3**	µg/100g	22	37	47	36	29	34±9
	**Vitamin A_1**	µg/100g	7.0	8.0	14	11	11	10±3
	**Calcium**	mg/100g	2800	3200	3300	2900	3000	3000±210
	**Iron**	mg/100g	15	20	23	18	18	19±3
	**Zinc**	mg/100g	5.1	5.3	5.2	4.8	5.1	5.1±0.2
	**Selenium**	µg/100g	210	290	260	250	200	240±37
	**Iodine**	µg/100g	110	190	150	140	120	142±28
**Microbial Quality**	**TCC aerob**	log CFU/g	4.36	4.55	4.90	3.98	5.33	4.62±0.52
	**TCC anaerob**	log CFU/g	3.76	4.04	4.40	2.78	5.29	4.05±0.92
	**Coliform**	log CFU/g	2.04	4.01	1.78	<LOD	3.89	2.93±1.19
**Contaminants**	**PAH4**	µg/kg	520	320	310	520	420	418±103
	**Cadmium**	mg/kg	0.16	0.18	0.17	0.18	0.24	0.19±0.03
	**Lead**	mg/kg	0.10	0.061	0.075	0.08	0.16	0.095±0.039
	**Mercury**	mg/kg	0.050	0.034	0.029	0.030	0.027	0.034±0.009
	**Arsenic**	mg/kg	7.0	14	10	11	6.8	9.8±3.0

[a] Values are given as mean ± SD. [b] One composite sample was analyzed comprising 73 specimens. [c] One composite sample was analyzed comprising 36 specimens. [d] One composite sample was analyzed comprising 46 specimens. [e] One composite sample was analyzed comprising 33 specimens. [f] One composite sample was analyzed comprising 57 specimens. LOD: Limit of detection. SFA - saturated fatty acids; MUFA - monounsaturated fatty acids; PUFA - polyunsaturated fatty acids; EPA - eicosapentaenoic acid; DHA - docosahexaenoic acid; TCC – total colony count; PAH4 – sum of benz(a)anthracene, benzo(a)pyrene, benzo(b)fluoranthene and Chrysene.

S2D Table: Analytical values for selected nutrients, microbial quality and contaminant concentration in composite samples (n=3) of smoked African moonfish (including head, skin, viscera and bones) from selected cities in Ghana (mean±SD)[a]

		Unit	Accra[b]	Techiman[c]	Kumasi[d]	Mean all markets
Nutrients	Protein	g/100g	69	68	66	67±2
	Total Fat	g/100g	6.6	7.2	5.3	6.3±0.9
	Sum SFA	g/100g (%)	2.1 (36)	2.2 (40)	1.8 (39)	2.0±0.2 (38)
	Sum MUFA	g/100g (%)	0.99 (17)	0.96 (18)	0.81 (18)	0.92±0.10 (18)
	Sum PUFA	g/100g (%)	2.0 (36)	2.0 (37)	1.7 (37)	1.9±0.2 (37)
	EPA	g/100g (%)	0.36 (6)	0.39 (7)	0.26 (6)	0.34±0.07 (6)
	DHA	g/100g (%)	1.2 (21)	1.0 (19)	0.94 (21)	1.0±0.1 (20)
	Vitamin B12	µg/100g	13	15	13	14±1
	Vitamin D_3	µg/100g	13	7.0	7.0	9.0±3.4
	Vitamin A_1	µg/100g	300	330	240	290±46
	Calcium	mg/100g	5300	5500	5600	5500±150
	Iron	mg/100g	64	43	44	50±12
	Zinc	mg/100g	4.0	5.9	4.8	4.9±1.0
	Selenium	µg/100g	160	170	190	170±15
	Iodine	µg/100g	260	210	230	230±25
Microbial Quality	TCC aerob	log CFU/g	5.06	4.58	5.14	4.93±0.30
	TCC anaerob	log CFU/g	4.18	3.67	4.08	3.98±0.27
	Coliform	log CFU/g	<LOD	2.64	2.74	2.69±0.07
Contaminants	PAH4	µg/kg	480	510	340	443±91
	Cadmium	mg/kg	0.080	0.043	0.072	0.065±0.019
	Lead	mg/kg	0.12	0.27	0.32	0.24±0.10
	Mercury	mg/kg	0.031	0.047	0.057	0.045±0.013
	Arsenic	mg/kg	4.1	6.1	6.8	5.7±1.4

[a] Values are given as mean ± SD. [b] One composite sample was analyzed comprising >100 specimens. [c] One composite sample was analyzed comprising >40 specimens. [d] One composite sample was analyzed comprising >85 specimens. LOD: Limit of detection. SFA - saturated fatty acids; MUFA - monounsaturated fatty acids; PUFA - polyunsaturated fatty acids; EPA - eicosapentaenoic acid; DHA - docosahexaenoic acid; TCC – total colony count; PAH4 – sum of benz(a)anthracene, benzo(a)pyrene, benzo(b)fluoranthene and Chrysene.

S2E Table: Analytical values for selected nutrients, microbial quality and contaminant concentration in composite samples (n=3) of whole smoked or dried West African pygmy herring (including head, skin, viscera and bones) from selected cities in Ghana (mean±SD)[a]

		Unit	Accra[b,c]	Techiman[d,e]	Bolgatanga[e,f]	Mean all markets
Nutrients	Protein	g/100g	71	65	67	67±3.1
	Total Fat	g/100g	8.4	14	15	12±3.6
	Sum SFA	g/100g (%)	3.5 (39)	3.7 (39)	4.9 (38)	4.0±0.8 (39)
	Sum MUFA	g/100g (%)	1.9 (21)	3.0 (31)	3.8 (29)	2.9±1.0 (27)
	Sum PUFA	g/100g (%)	3.1 (35)	2.5 (26)	3.7 (29)	3.1±0.6 (30)
	EPA	g/100g (%)	0.34 (4)	0.22 (2)	0.32 (2)	0.29±0.06 (3)
	DHA	g/100g (%)	1.0 (12)	0.60 (6)	0.69 (5)	0.77±0.22 (8)
	Vitamin B12	µg/100g	14	23	11	16±6
	Vitamin D_3	µg/100g	29	10	14	18±10
	Vitamin A_1	µg/100g	8.0	60	50	39±28
	Calcium	mg/100g	2200	2900	2800	2600±380
	Iron	mg/100g	180	21	32	78±89
	Zinc	mg/100g	18	13	13	15±3
	Selenium	µg/100g	110	81	92	94±15
	Iodine	µg/100g	66	210	110	130±74
Microbial Quality	TCC aerob	log CFU/g	NA	5.62	6.67	6.15±0.74
	TCC anaerob	log CFU/g	NA	NA	6.61	NA
	Coliform	log CFU/g	NA	4.02	2.21	3.12±1.28
Contaminants	PAH4	µg/kg	9	370	1300	560±666
	Cadmium	mg/kg	0.018	0.011	0.015	0.015±0.004
	Lead	mg/kg	1.3	0.093	0.53	0.64±0.61
	Mercury	mg/kg	0.15	0.37	0.15	0.22±0.13
	Arsenic	mg/kg	1.6	0.64	0.60	0.95±0.57

[a] Values are given as mean ± SD. [b] One composite sample was analyzed comprising >200 specimens. [c] Dried fish. [d] One composite sample was analyzed comprising >100 specimens. [e] Smoked fish. [f] One composite sample was analyzed comprising >100 specimens. NA: Not available. SFA - saturated fatty acids; MUFA - monounsaturated fatty acids; PUFA - polyunsaturated fatty acids; EPA - eicosapentaenoic acid; DHA - docosahexaenoic acid; TCC – total colony count; PAH4 – sum of benz(a)anthracene, benzo(a)pyrene, benzo(b)fluoranthene and Chrysene.

S2F Table: Analytical values for selected nutrients, microbial quality and contaminant concentration in composite samples (n=5) of salted tilapia (including head, skin and bones) from selected cities in Ghana (mean±SD)[a]

		Unit	Accra	Techiman	Tamale	Kumasi	Bolgatanga	Mean all markets
Nutrients	Protein	g/100g	33	30	30	28	38	32±3.9
	Total Fat	g/100g	6.0	5.9	6.9	8.4	11	7.6±1.9
	Sum SFA	g/100g (%)	2.8 (40)	2.4 (32)	3.3 (44)	3.0 (39)	3.2 (40)	2.9±0.4 (39)
	Sum MUFA	g/100g (%)	2.0 (29)	1.5 (28)	2.1 (27)	2.3 (30)	2.4 (31)	2.1±0.4 (29)
	Sum PUFA	g/100g (%)	1.7 (26)	1.4 (24)	1.6 (21)	1.9 (24)	1.7 (22)	1.7±0.2 (23)
	EPA	g/100g (%)	0.04 (0.5)	0.04 (0.6)	0.03 (0.3)	0.06 (0.8)	0.04 (0.5)	0.04±0.01 (0.5)
	DHA	g/100g (%)	0.25 (4)	0.15 (2)	0.10 (1)	0.22 (3)	0.17 (2)	0.18±0.06 (2)
	Vitamin B12	µg/100g	14	12	9.8	10	8.3	11±2
	Vitamin D_3	µg/100g	8.0	17	12	17	10	13±4
	Vitamin A_1	µg/100g	19	<LOQ	<LOQ	<LOQ	<LOQ	<LOQ
	Calcium	mg/100g	3400	2300	NA	2300	2600	2700±520
	Iron	mg/100g	7.8	16	9.4	7.4	9.1	9.9±3.5
	Zinc	mg/100g	5.2	3.7	4.3	3.8	4.4	4.3±0.6
	Selenium	µg/100g	38	28	27	31	42	33±7
	Iodine	µg/100g	46	37	41	64	57	49±11
Microbial Quality	TCC aerob	log CFU/g	4.07	3.91	3.85	3.97	5.37	4.23±0.64
	TCC anaerob	log CFU/g	4.75	NA	3.64	3.61	5.72	4.43±0.64
	Coliform	log CFU/g	<LOD	<LOD	<LOD	<LOD	6.23	<LOD
Contaminants	PAH4	µg/kg	7.0	1.7	3.3	1.0	24	7.4±9.6
	Cadmium	mg/kg	<LOQ	<LOQ	<LOQ	<LOQ	<LOQ	<LOQ
	Lead	mg/kg	<LOQ	<LOQ	<LOQ	0.21	<LOQ	<LOQ
	Mercury	mg/kg	0.031	<LOQ	<LOQ	<LOQ	<LOQ	<LOQ
	Arsenic	mg/kg	<LOQ	0.086	0.10	0.11	0.11	0.095±0.017

[a] Mean ± SD. [b] One composite sample was analyzed comprising 73 specimens. [c] One composite sample was analyzed comprising 34 specimens [d] One composite sample was analyzed comprising 35 specimens. [e] One composite sample was analyzed comprising 50 specimens [f] One composite sample was analyzed comprising 22 specimens. LOD: Limit of detection; LOQ: Limit of quantification. SFA - saturated fatty acids; MUFA - monounsaturated fatty acids; PUFA - polyunsaturated fatty acids; EPA - eicosapentaenoic acid; DHA - docosahexaenoic acid; TCC – total colony count; PAH4 – sum of benz(a)anthracene, benzo(a)pyrene, benzo(b)fluoranthene and Chrysene.
